# Microbiological contamination in androgens from the black market

**DOI:** 10.1186/s12954-025-01359-w

**Published:** 2025-12-05

**Authors:** T. J. Verdegaal, P. Bond, M. J. H. M. Jansen, M. Peijs, M. Huyers, J. J. J. M. Stohr, W. de Ronde, D. L. Smit

**Affiliations:** 1 Department of Internal Medicine, Spaarne Gasthuis, Boerhaavelaan 22, 2035 RC Haarlem, North Holland Netherlands; 2 Department of Performance and Image-Enhancing Drugs Research, Android Health Clinic, Leidse Rijn 31, De Meern, 3454 PZ Utrecht, Netherlands; 3 Department of Internal Medicine, Elisabeth-TweeSteden Hospital, Doctor Deelenlaan 5, 5042 AD Tilburg, North Brabant Netherlands; 4 Department of Medical Microbiology and Immunology, Elisabeth- TweeSteden Hospital, Doctor Deelenlaan 5, 5042 AD Tilburg, North Brabant Netherlands

**Keywords:** Microbiological contamination, Anabolic steroids, Androgen abuse, Performance and image-enhancing drugs

## Abstract

**Background:**

Androgens from the black market are often produced in clandestine laboratories without adherence to hygienic manufacturing processes. Consequently, users may face an increased risk of developing injection-related infections caused by microbiological contamination. This study aimed to determine the presence of microbiological contamination in androgens from the black market.

**Methods:**

Characteristics of submitted androgen product were registered. Androgen products were submitted to aerobic and anaerobic bacterial culture using direct inoculation of agar plates and enrichment using BD BACTEC™ Peds Plus Medium blood culture bottles. Participants who submitted products were monitored for injection-related infections.

**Results:**

Bacterial contamination was detected in two of 22 used multidose vials (9%) and one of 41 unused ampules and multidose vials (2%). Identified species included *Bacillus spp.*, *Staphylococcus epidermidis*, *Staphylococcus warneri*, *Staphylococcus saprophyticus* and *Micrococcus luteus*. None of the participants developed injection-related infections linked to contaminated products.

**Conclusion:**

Both used and unused androgens from the black market can contain bacteria. This can put users at risk for serious injection-related health problems such as abscesses. This study underlines the importance of warning androgen users about these underrecognized injection-related risks.

**Trial registration:**

Study number: NL77191.028.21. Registration date: 2 June 2021.

## Background

Anabolic androgenic steroids are a class of compounds that includes testosterone and other androgens. These androgens are widely abused for their muscle-building effects. Most users administer androgens and other image- and performance enhancing drugs (IPEDs) via intramuscular or subcutaneous injections, often once or multiple times per week. Therefore, in addition to systemic health risks such as hepatotoxicity and cardiovascular disease [1], users are at an increased risk for injection-related health problems such as local tissue injury and injection-related infections [2, 3]. Indeed, approximately half of androgen users report to have experienced symptoms such as redness, tenderness and swelling at an injection site [4, 5]. Studies reported the prevalence of a user having had an abscess, sore or open wound at an injection site to be 4% [3, 6] to 7% [4].

Case reports have documented androgen users contracting infections with bacterial pathogens [7–15] and blood-borne viruses such as human immunodeficiency virus (HIV) [16, 17], hepatitis B [17] and hepatitis C [18]. In most reports, non-sterile injection techniques, needle reuse and sharing were the suspected cause for the infection. In recent years, such high-risk practices have become less common, likely due to easier access to sterile injection equipment through pharmacies and online vendors, increased harm reduction efforts, and widespread dissemination of safe injection guidance [19].

However, injection-related infections may also result from microbiological contamination of the product itself. Black-market androgens are typically produced in clandestine laboratories that may lack adequate facilities, trained personnel, and adherence to good manufacturing practices [20]. The imprecise and careless process of producing underground androgens is evidenced by the qualitative and quantitative impurities of androgens in a substantial portion of androgen products [3, 20]. Substandard manufacturing practices increase the risk for microbiological contamination during various stages of the production, including the use of contaminated raw materials, to inadequate hygiene during production, packaging or transport.

The risk is further amplified because underground products are often sold in multidose 10 mL vials, which are accessed repeatedly, increasing opportunities for contamination. Only one previous study has reported contamination of parenteral androgens confirmed with microbiological culture techniques, reporting exclusively on unused products [21]. No data are available on contamination rates in used products. To address this gap, the present study examines the prevalence of microbiological contamination in both used and unused parenteral androgens from the Dutch black market.

## Methods

Men of at least 18 years old taking part in a prospective cohort study investigating the efficacy of harm reduction in androgen use were asked if they were willing to provide used and/or unused, i.e. sealed, multidose vials or ampules containing androgens for this analysis. These men had given informed consent to participate in a cohort study, that was brought to the attention of the bodybuilding community by social media, if they intended to perform an androgen cycle on short notice. During the cohort study, participants visited the hospital three times where they received a harm reduction intervention. The study protocol and informed consent was approved by the Institutional Review Board (study number: NL77191.028.21) in June 2021 after which inclusion of participants started. All included participants signed informed consent for their participation. All procedures were performed in accordance with relevant guidelines and regulations.

The participants retrieved the underground androgen products through their usual black-market channels, predominantly through internet webshops or local androgen dealers. Participants did not receive a reward or reimbursement for their enrolment in this trial or for their donation of androgen products. The participants donated the samples during the hospital visits that were part of the cohort study.

The first 63 donated androgen products containing a minimum of 1 mL of oil were included in this analysis. For every androgen product included in the study the following characteristics were registered: brand, androgen subtype (e.g. testosterone, nandrolone, trenbolone) as declared on the product label, type of vial (glass ampule or multi-use glass container), previous usage (yes or no) and number of times used. Participants were monitored for the occurrence of injection related infections via questionnaire during hospital visits. On the day the androgen product was donated to the investigators, the androgen products were brought to the department of medical microbiology and immunology, where microbiological analysis was started.

### Microbiological methods

Fluid from every androgen product was inoculated on different media and incubated as follows; 0.1 mL was inoculated onto sheep blood and chocolate agar and incubated aerobically at 37 °C for 4 days; 0.1 mL was inoculated onto Wilkins agar and incubated anaerobically at 37 °C for 4 days; 0.1 mL was inoculated in BD BACTEC™ Peds Plus Medium (BD Diagnostics, MD, USA) supplemented with 2 mL of BD BACTEC™ FOS Culture Supplement (BD Diagnostics, MD, USA) and incubated aerobically for 5 days at 37 °C in the BD BACTEC™ FX blood culture system (BD Diagnostics, MD, USA). When growth was detected in the BD BACTEC™ FX blood culture system, 0.01mL of the grown suspension was inoculated on BD™ Columbia CNA agar (BD Diagnostics, MD, USA) with 5% sheep blood, chocolate agar and MacConkey agar plates which were subsequently incubated aerobically at 37 °C for 4 days. Species identification of grown colonies was performed using the Bruker MALDI Biotyper™ (BD Diagnostics, MD, USA), and antimicrobial susceptibility testing was performed using the PhoenixTM platform (BD Diagnostics, MD, USA) and EUCAST breakpoints v.14.0.

## Results

In total 63 androgen products were collected from 31 participants. Samples originated from 31 different brands and comprised seven different androgen subtypes as declared on the product label. Not all participants were willing to donate androgen products, being especially unwilling to donate unused multidose vials. The primary reason for non-donation was financial as products could not be returned once used for microbiological testing and no reimbursement was provided, making donation costly for participants. A secondary reason was that some participants had only purchased the amount of androgens required for their planned cycle and would therefore need to obtain additional products to donate a sample. As this would require renewed interaction with black market suppliers, several participants were reluctant to do so solely to donate for research purposes. In contrast, participants were generally willing to provide any recently used vials.

Of the 63 androgen products analyzed, three (5%) showed evidence of bacterial contamination. Two of 22 used multidose vials (9%) demonstrated bacterial growth on culture, each showing few colonies (defined as 1–15 colonies). The third contaminated product, one of 41 unused ampules and multidose vials (2%) yielded a positive result following enrichment in the BD BACTEC™ blood culture system. The characteristics of the contaminants are described in Table 1.

**Table 1:** Identified bacterial species in counterfeit androgens

Positive sample	Use status	Identified bacteria
Multidose vial 1	Used	1. *Bacillus spp.*, 2. *Staphylococcus epidermidis*
Multidose vial 2	Used	1. *Micrococcus luteus*, 2. *Staphylococcus warneri*,
Ampule	Unused	1. *Staphylococcus saprophyticus*

On average, used multidose vials were broached 8 times by users (range 1–66). The two positive used androgen products were broached 4 and 7 times, respectively. Some participants reported-injection related complaints such as persistent muscle pain and swelling from using the androgen product which they had supplied, but these vials did not contain microbiological contamination. The participant that supplied the two positive multidose vials had not experienced any symptoms from the use of those products.

## Discussion

This study is the first to systematically investigate microbiological contamination in used androgen products from the black market. Contamination with commensal bacteria was detected in several vials. In the unused vial, the contamination likely reflects poor manufacturing hygiene. However, the majority of unused androgen products was free of bacterial contamination. This indicates that, despite their illicit origin, these products may be produced under reasonably hygienic manufacturing procedures, or that certain physicochemical characteristics of androgen products suppress bacterial growth. Relevant factors may include the addition of excipients with antimicrobial activity such as benzyl alcohol [22], as well as the low water content and high viscosity of the carrier oil. In used vials, contamination may also have resulted from unhygienic injection practices, including repeated vial access and insufficient aseptic technique. Given that multidose vials—commonly containing 10 mL of oil-based androgen solution—are often accessed multiple times, it is plausible that users occasionally introduce bacteria themselves.

The detection of bacteria in both used and unused vials in this study suggests that exposure to contaminated androgen products may be more common than previously acknowledged. The absence of injection-related symptoms in the participant whose vials tested positive provides an indication that contamination with low-virulence bacteria does not necessarily result in clinical infection or local inflammation. However, low-virulence bacteria can cause notable complications such as injection-site abscesses following androgen administration [11]. Most of the bacterial species identified in this study have previously been associated with various clinical infections including bacteremia, sepsis and endocarditis, occurring predominantly in immunocompromised but sporadically also in immunocompetent patients [23–27]. Though serious infection may be relatively rare in immunocompetent users, this risk is likely to be amplified by the many repeated injections and multiple vials typically used over the course of many cycles [28], which compounds the cumulative risk over time.

While only bacteria of low virulence were identified in this study, the detection of contamination in several samples raises the possibility that black-market androgens may occasionally harbour more virulent organisms. This possibility is supported by several case reports describing bacterial infection following androgen injections in which cultured organisms include *Staphylococcus aureus* [9, 14], pseudomonas [12] and atypical mycobacteria [7, 13, 29]. Importantly, these infections do not necessarily imply contamination of the product itself. Alternative mechanisms include direct introduction of skin bacteria at the injection site or when post-injection intramuscular microtraumatic lesions become infected due to bacteremia from a *porte d’entrée* situated elsewhere (e.g. oral cavity, nasal passages, skin lesions).

Several limitations should be acknowledged. The sample size of analyzed products was modest, which precludes precise estimation of the true prevalence of contamination among black market androgens. However, in the absence of any prior prospective data on contamination of used androgens, these results provide useful preliminary insight into the occurrence and likely frequency of contamination in androgens. In addition, as sample donation was voluntary, selection bias cannot be excluded. The lack of reimbursement for donated material may have disproportionately discouraged individuals with more limited socioeconomic means. Therefore, the findings should be interpreted as indicative rather than representative of the broader population of androgen users.

A further consideration is that all participants that donated androgens were enrolled in a harm-reduction intervention, which may have influenced the observed contamination rates. The intervention addressed several aspects of androgen use, including dosage and duration of use, compound selection, product contamination and general injection hygiene. This individualized counseling and discussion of health risks may have heightened participants’ overall awareness and may have encouraged more cautious behaviors. Without a non-intervention control group, the magnitude of this influence cannot be determined, but it remains plausible that increased vigilance contributed to a lower observed prevalence of contamination.

While efforts to reduce androgen abuse must continue to focus on the systemic dangers of these substances, greater awareness is needed for the harms related to injection-related harms and contamination risks, even when sterile needles are used. Importantly, although oral androgens do not carry injection-related risks, it remains important to dissuade users from transitioning to oral androgens, as they are considered to be more harmful than parenteral androgens due to their increased hepatotoxic potential and disruption of lipid profiles [1].

## Conclusion

This study adds to the evidence that illicitly produced androgens may be produced under poor hygienic conditions, as bacterial commensals were detected in both unused and used black-market products. Unhygienic injection techniques, such as repeated access to multidose vials, may further contribute to contamination of androgen products. Given the large number of cycles typically undertaken by users, exposure to contaminated products is likely recurrent, placing individuals at risk for injection-related complications, such as abscesses, that can be severe enough to lead to hospital admission. These findings highlight the need to warn androgen users about these risks.

## Data Availability

The data that support the findings of this study are available from the corresponding author, TV, upon reasonable request.
